# A Practical Treatise on Inflammation, Ulceration, and Induration of the Neck of the Uterus, &c., &c.

**Published:** 1847-08

**Authors:** 


ARTICLE XIII.

*A Practical Treatise on Inflammation, Ulceration, and Induration of the Neck of the Uterus, with Remarks on Leucorrhœa and Prolapsus Uteri, as Symptoms of Uterine Disease.* By James Henry Bennett, M.D., Licentiate of the Royal College of Physicians, &c., &c. Philadelphia: Lea & Blanchard. 1847. pp. 146. (From the Publishers, by Joseph Keen, Chicago.)

Nothing could be more opportune than this little work of Dr. Bennet; for if, as he states, English practitioners seem unconscious of the existence of the diseases of which he treats, not only is this true of America, but the symptoms they produce are generally taken for independent diseases, and treated by mechanical means alone. Charlatanism, taking advantage of this popular opinion, both in and out of the profession, has found its interest in inventing pessaries, supporters, and bandages of all sorts, and patients treated by these are frequently for years affected with nervous and hysterical affections, who might once have been cured by appropriate treatment. On every account this book is to be recommended—the subject, the size, the matter, and the manner are all good. We propose in our next to give a very full analysis of its contents. D. B.

